# Supplementary material for: Sublinear domination and core–periphery networks
Source: Sci Rep. 2021 Jul 30;11:15528. doi: 10.1038/s41598-021-94105-8 (PMC8324891; doi:10.1038/s41598-021-94105-8)
Supplement: Supplementary file 1 — Supplementary Information. [file 41598_2021_94105_MOESM1_ESM.pdf]

Sublinear Domination and Core–periphery Networks
Supplementary Material

Marios Papachristou
Cornell University
papachristoumarios@cs.cornell.edu

The current notebook serves as a static version of the supplementary material needed to reproduce the figures presented in the original paper. The interactive notebook can also be found here.

1 Simulations

We first perform the necessary imports.

[ ]:
```
import numpy as np
import networkx as nx
import collections
import matplotlib.pyplot as plt
import pandas as pd
import seaborn as sns
import matplotlib.cm as cm

sns.set_theme()

LARGE_SIZE = 16
plt.rc('axes', labelsize=LARGE_SIZE)
plt.rc('axes', titlesize=LARGE_SIZE)
```

1.1 IGAM/IGAM2 Model

We now present the implementation for generating instances of the IGAM model. More specifically, the `generate_igam2` function is responsible for generating IGAM2 instances with

- height H
- core height $H_0 < H$
- fanout $b \in \mathbb{Z}_{\geq 2}$
- Scale parameters c_1, c_2 with $1 < c_1 < c_2 < b$.

The law for generating the **IGAM2** model is given as

$$g(u,v) = \begin{cases} c_2^{-1-\min\{h(u),h(v)\}} & \max\{h(u),h(v)\} > H_0 \\ c_1^{-1-\min\{h(u),h(v)\}} & \max\{h(u),h(v)\} \leq H_0 \end{cases}$$

Letting $c_1 = c_2$ the parameter H_0 becomes redundant and the law reduces to the **IGAM** law of edge generation

$$f(u,v) = c^{-1-\min\{h(u),h(v)\}}$$

Below we give a plot of IGAM with parameters $H = 5$, $b = 2$ and $c = c_1 = c_2 = 1.5$. We also give plots of the pagerank vector computed for the particular instance with values sorted in ascending order.

[ ]:
```
def generate_igam2(H=5, b=2, c1=1.5, c2=1.5, H0=2):
  G = nx.generators.classic.balanced_tree(r=b, h=H)
  n_i = b**H
  temp = np.log(b / c1)

  black = collections.defaultdict(bool)
  x_axis, y_axis = [], []
  edges_int = list(G.edges())
  edges_rnd = []
  nodelist = collections.defaultdict(list)

  height_histogram = np.zeros(H + 1)

  for u in G:
    for v in reversed(list(G.nodes())):
      hu = np.floor(np.log(u + 1) / np.log(b))
      hv = np.floor(np.log(v + 1) / np.log(b))
      un = np.random.uniform(low=0, high=1)
      nodelist[int(hu)].append(u)
      min_h = min(hu, hv)
      max_h = max(hu, hv)
      if max_h <= H0 + 1:
        if un <= c1**(-1 - min_h):
          G.add_edge(u, v)
          edges_rnd.append((u, v))
          height_histogram[int(hu)] += 1
          height_histogram[int(hv)] += 1
      else:
        if un <= c2**(-1 - min_h):
          G.add_edge(u, v)
          edges_rnd.append((u, v))
          height_histogram[int(hu)] += 1
          height_histogram[int(hv)] += 1
```

```
  G.remove_edges_from(edges_int)

  for u in G:
    black[u] = True
    for v in G.neighbors(u):
      black[v] = True
      x_axis.append((u + 1) / len(G) * 100)
      y_axis.append(len(black) / len(G) * 100)

  x_axis = np.array(x_axis)
  y_axis = np.array(y_axis)

  return G, x_axis, y_axis, height_histogram, nodelist, H, b, c1, c2, n_i

def plot_igam2(G, x_axis, y_axis, height_histogram, nodelist, H, b, c1, c2, n_i,␣
↪suffix=''):
  plt.figure(figsize=(8, 8))
  plt.title('Domination Curve')
  plt.ylabel('Percentage of dominated external nodes (linear)')
  plt.xlabel('Percentage of internal nodes included by order of height (log)')
  plt.plot(x_axis, y_axis)
  plt.savefig('iga_curve_general{}.png'.format(suffix))

  plt.figure()
  rank_range = np.arange(H + 1)
  height_histogram = np.log(height_histogram)
  plt.plot(rank_range, height_histogram, linewidth=0, marker='x', color='r',␣
↪label='Empirical Frequencies')
  plt.xlabel('Height')
  plt.ylabel('Number of Edges')
  p = np.polyfit(rank_range, height_histogram, deg=1)
  plt.plot(rank_range, p[0] * rank_range + p[1], label='$ y = {} x + {}$'.
↪format(round(p[0], 2), round(p[1], 2)))
  plt.legend()

  print('Diameter:', nx.diameter(G))
  print('Theoretical Diameter:', np.log(b) / np.log(b/c2))

  fig, ax = plt.subplots(1, 1, figsize=(15, 15))
  pos = nx.shell_layout(G)
  for h, nodes in nodelist.items():
    nx.draw_networkx_nodes(G, nodelist=nodes + [0], ax=ax, pos=pos,␣
↪node_color='b', node_size=50, alpha=(h / H))

  nx.draw_networkx_edges(G, edgelist=G.edges(), edge_color='r', pos=pos, alpha=0.
↪3)
  plt.savefig('iga_general{}.png'.format(suffix))
```

```
pr = nx.pagerank_numpy(G)
pr_values = np.array(list(pr.values()))[:, np.newaxis].T
sorted_pr_values = np.sort(pr_values)
plt.figure(figsize=(30, 10))
plt.imshow(sorted_pr_values)

plt.figure(figsize=(10, 10))
plt.imshow(nx.to_numpy_array(G))
plt.savefig('igam2_adj{}.png'.format(suffix))
```

[ ]:
```
G, x_axis, y_axis, height_histogram, nodelist, H, b, c1, c2, n_i =␣
 ↪generate_igam2()
plot_igam2(G, x_axis, y_axis, height_histogram, nodelist, H, b, c1, c2, n_i,␣
 ↪suffix='_simple_igam')
```

```
Diameter: 3
Theoretical Diameter: 2.4094208396532095
```

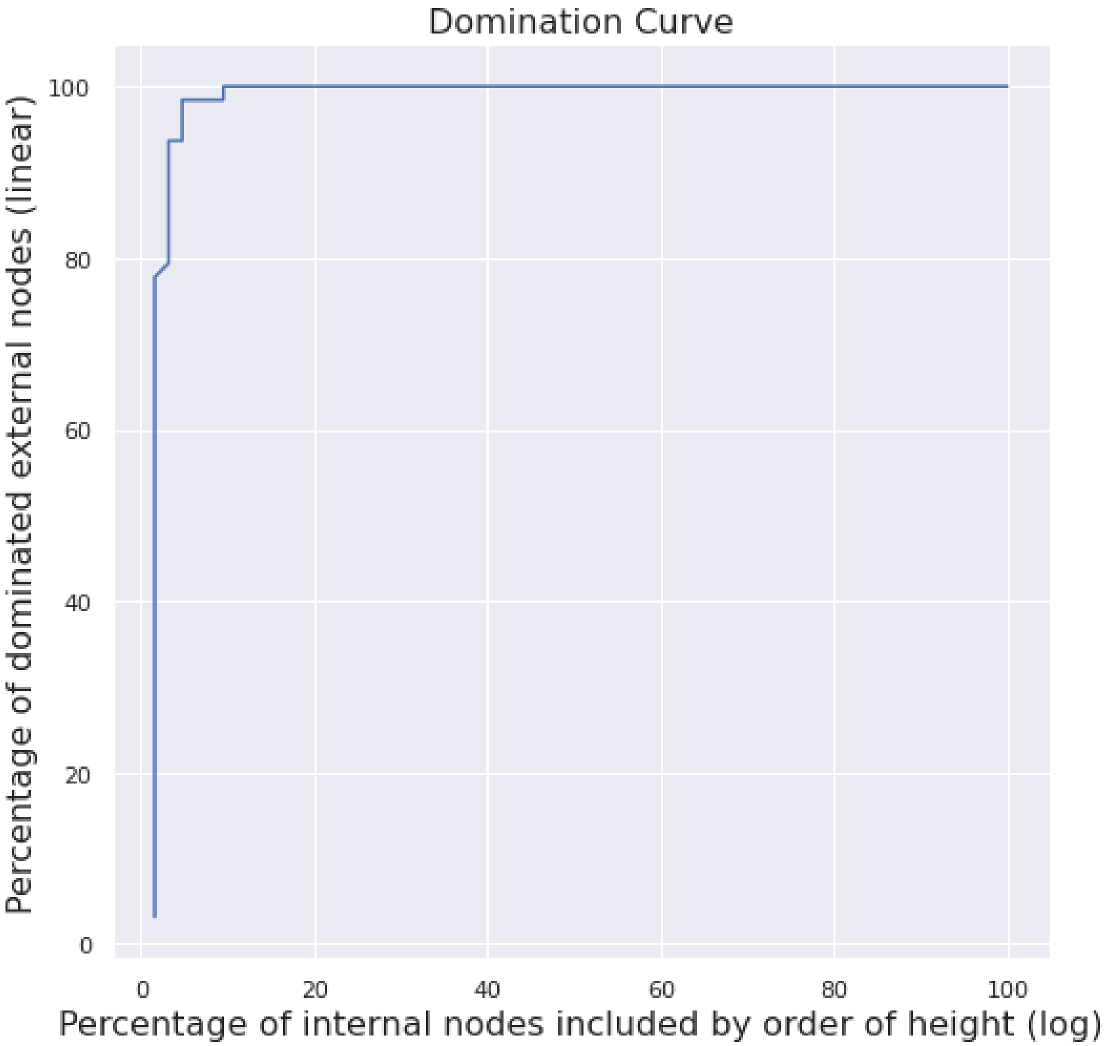

Supplementary Figure S1: Percentage of nodes dominated (linear) vs. number of nodes included by order of height (log) for the IGAM model with $H = 5,\ b = 2,\ c = 1.5$.

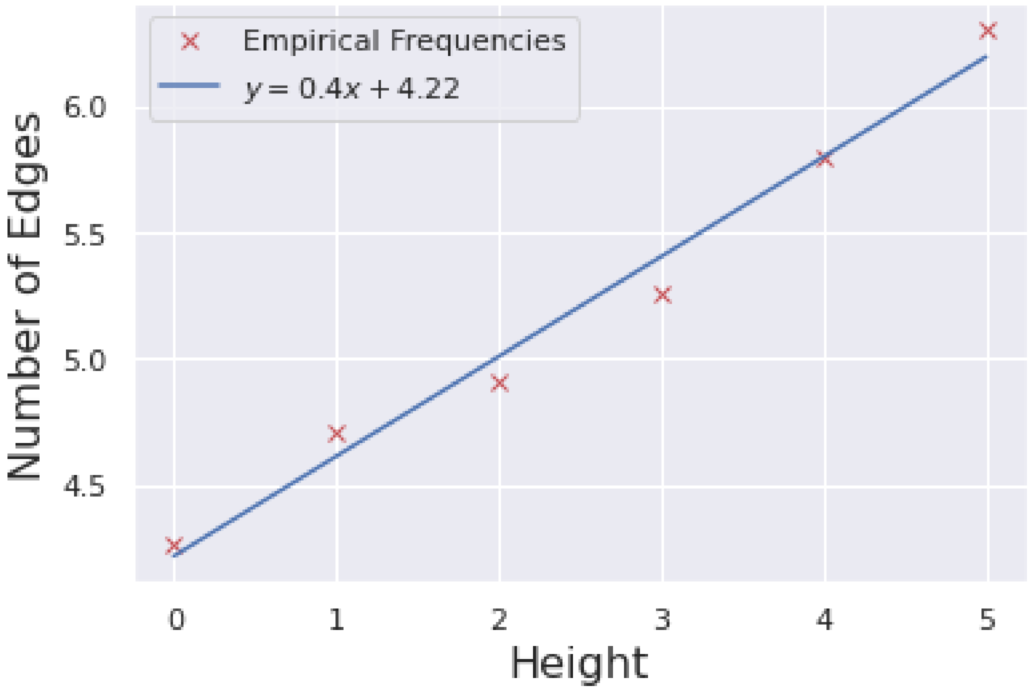

Supplementary Figure S2: Number of edges (log) with respect to height (linear) for the IGAM model with $H = 5,\ b = 2,\ c = 1.5$. Empirical frequencies and linear fit are provided.

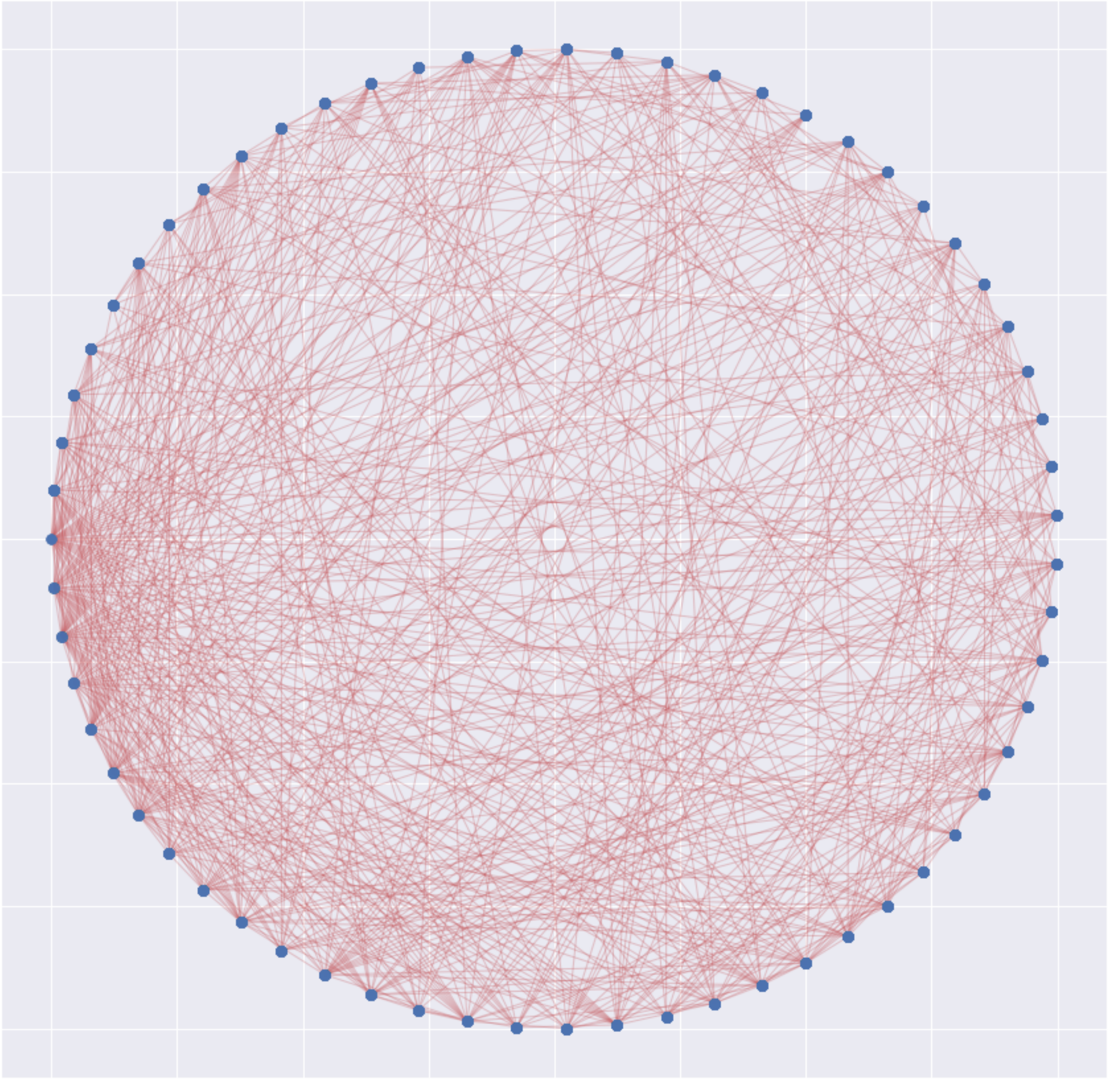

Supplementary Figure S3: Plot of IGAM instance with $H = 5,\ b = 2,\ c = 1.5$

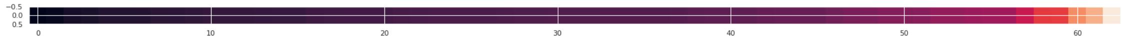

Supplementary Figure S4: Sorted Pageranks of IGAM instance with $H = 5,\ b = 2,\ c = 1.5$

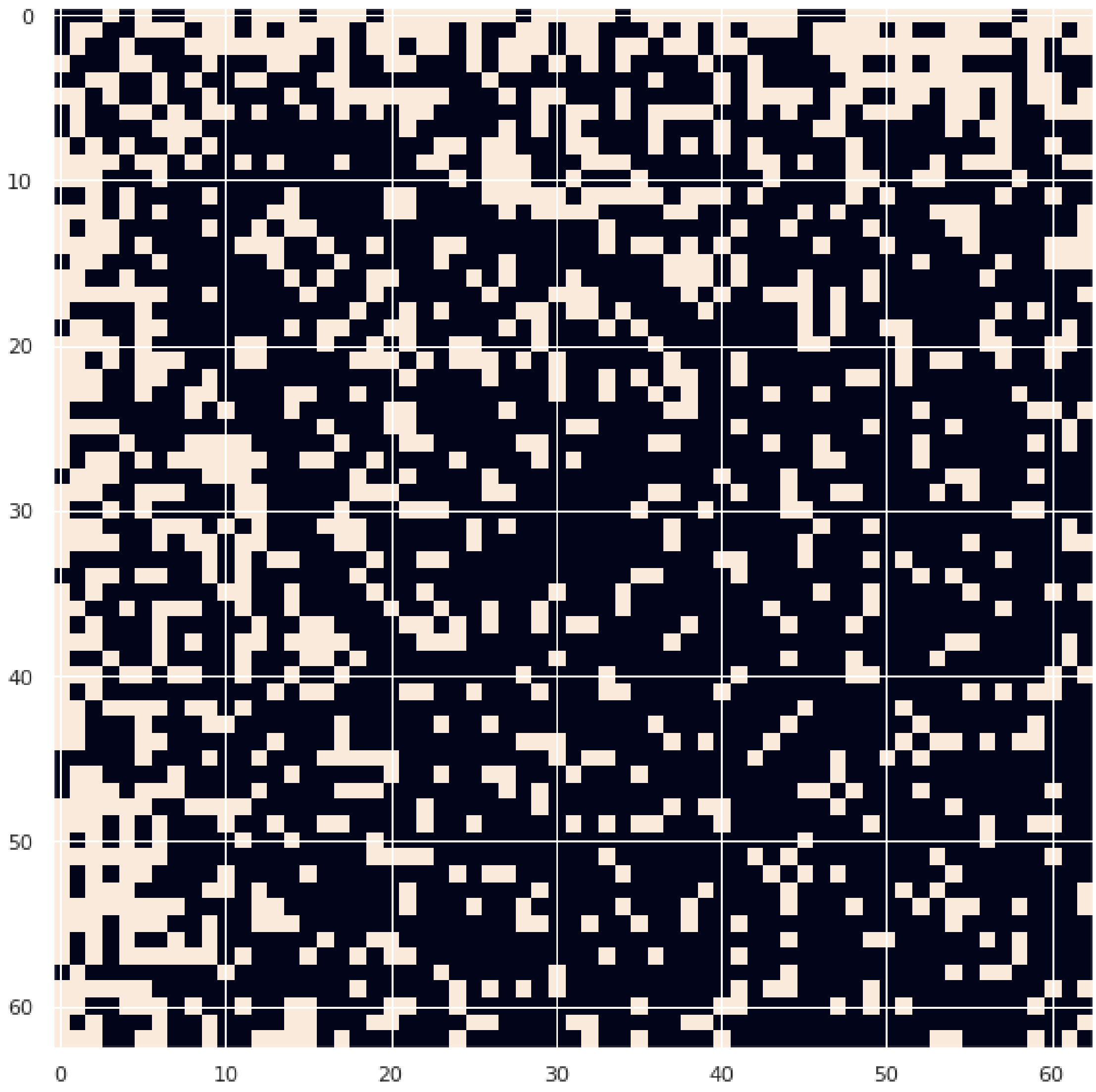

Supplementary Figure S5: Adjacency matrix of IGAM instance with $H = 5,\ b = 2,\ c = 1.5$

```
[ ]: G, x_axis, y_axis, height_histogram, nodelist, H, b, c1, c2, n_i =
       generate_igam2(H=10, b=2, c1=1.5, c2=1.5)
     plot_igam2(G, x_axis, y_axis, height_histogram, nodelist, H, b, c1, c2, n_i,
       suffix='_simple_igam')
```

```
Diameter: 3
Theoretical Diameter: 2.4094208396532095
```

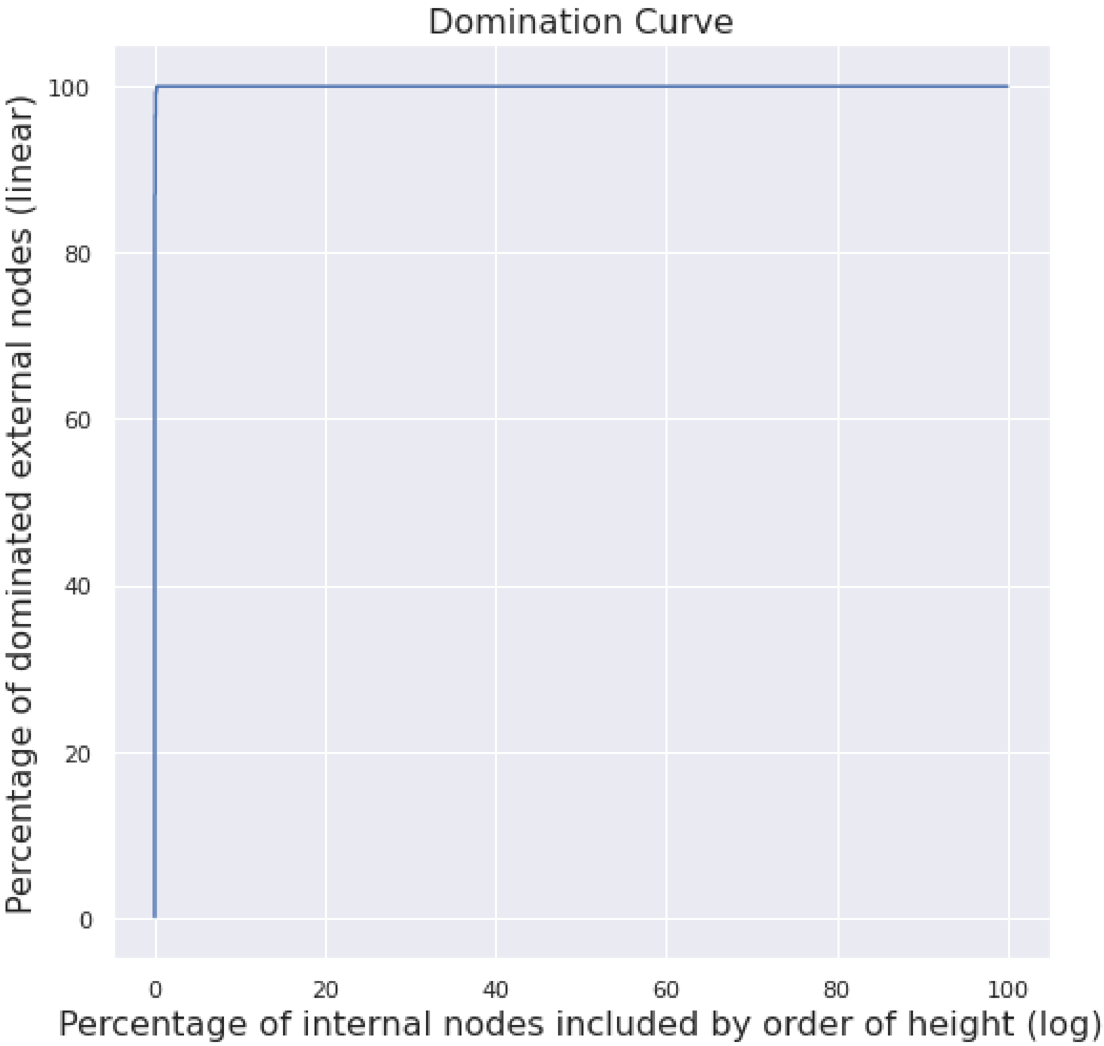

Supplementary Figure S6: Percentage of nodes dominated (linear) vs. number of nodes included by order of height (log) for the IGAM model with $H = 10,\ b = 2,\ c = 1.5$.

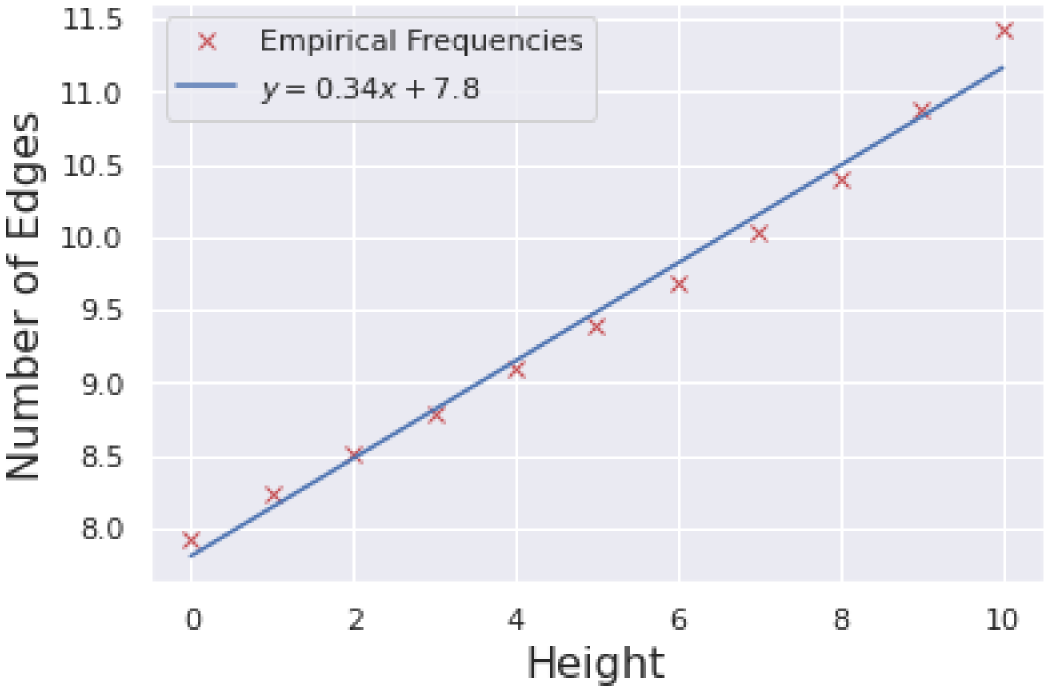

Supplementary Figure S7: Number of edges (log) with respect to height (linear) for the IGAM model with $H = 10,\ b = 2,\ c = 1.5$. Empirical frequencies and linear fit are provided.

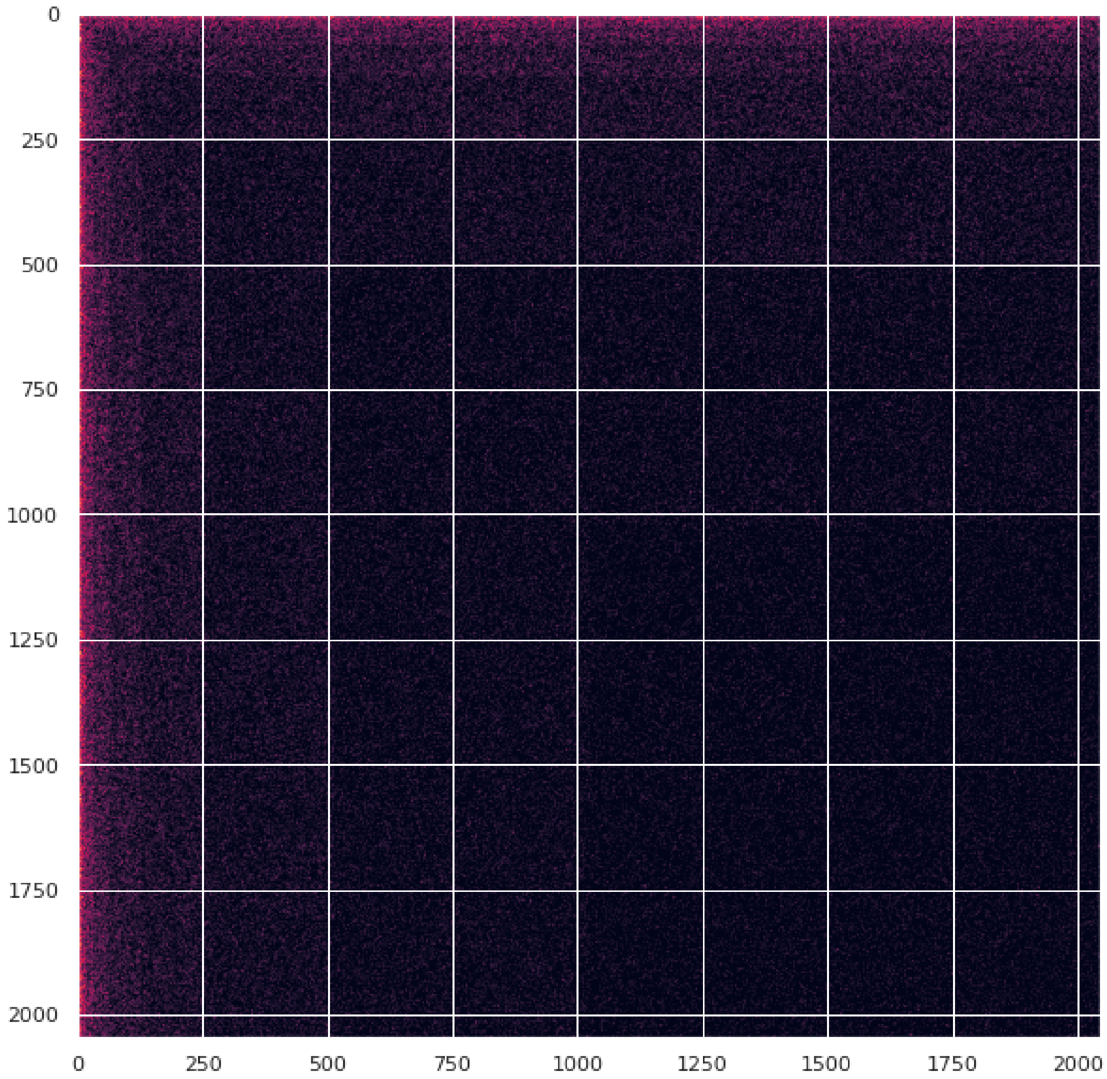

Supplementary Figure S8: Adjacency matrix of IGAM instance with $H = 10,\ b = 2,\ c = 1.5$

We also give a plot of IGAM2 with $b = 3$, $H = 6$, $H_0 = 2$, $c_1 = 1.5$ and $c_2 = 2.5$. The core-periphery pattern is evident by observing the adjacency matrix plot.

[ ]:
```
G, x_axis, y_axis, height_histogram, nodelist, H, b, c1, c2, n_i =␣
 ↪generate_igam2(H=6, b=3, c1=1.5, c2=2.5, H0=2)
plot_igam2(G, x_axis, y_axis, height_histogram, nodelist, H, b, c1, c2, n_i,␣
 ↪suffix='_simple_igam')
```

```
Diameter: 3
Theoretical Diameter: 6.025685102665476
```

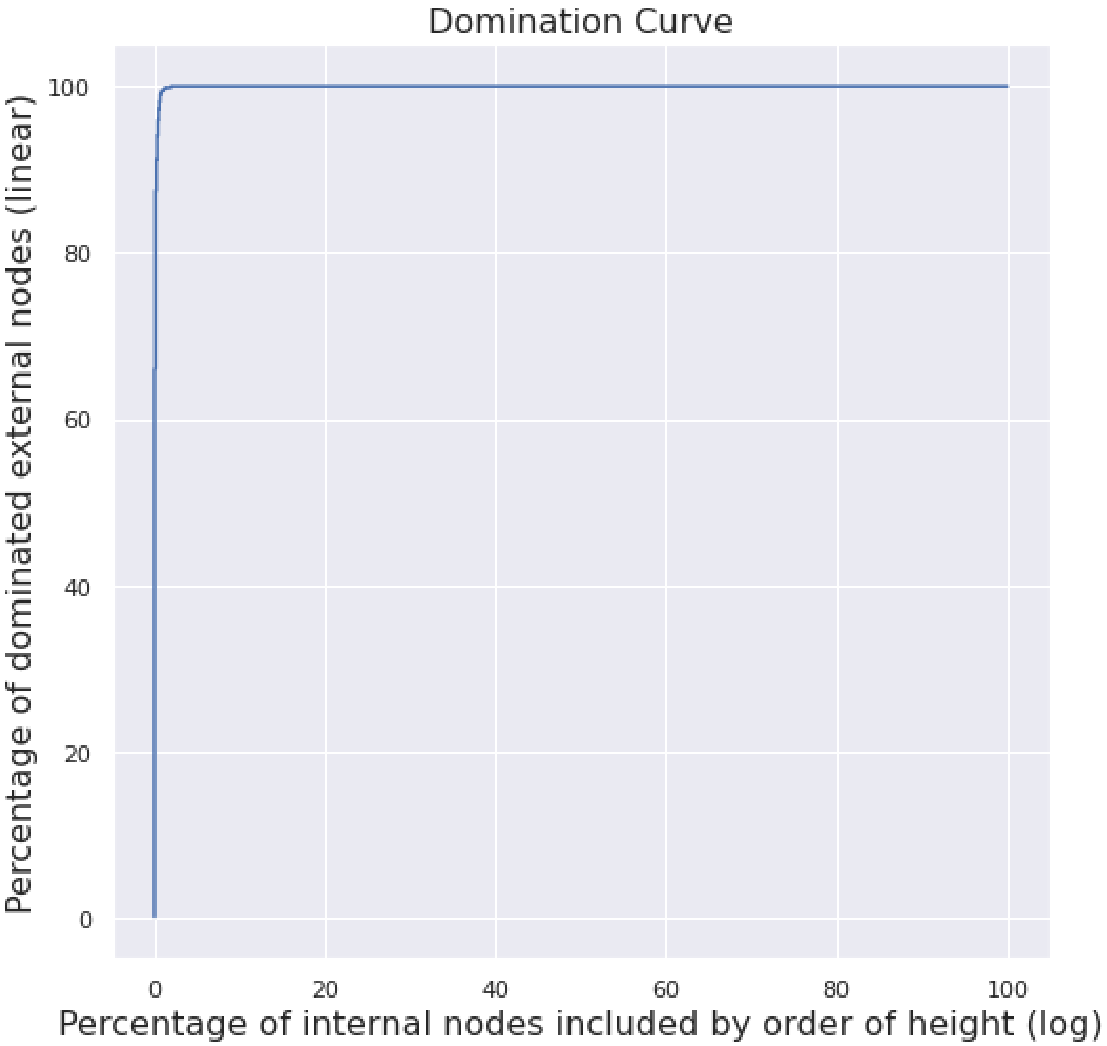

Supplementary Figure S9: Percentage of nodes dominated (linear) vs. number of nodes included by order of height (log) for the IGAM2 model with $H = 6,\ b = 3,\ c_1 = 1.5,\ c_2 = 2.5,\ H_0 = 2.$

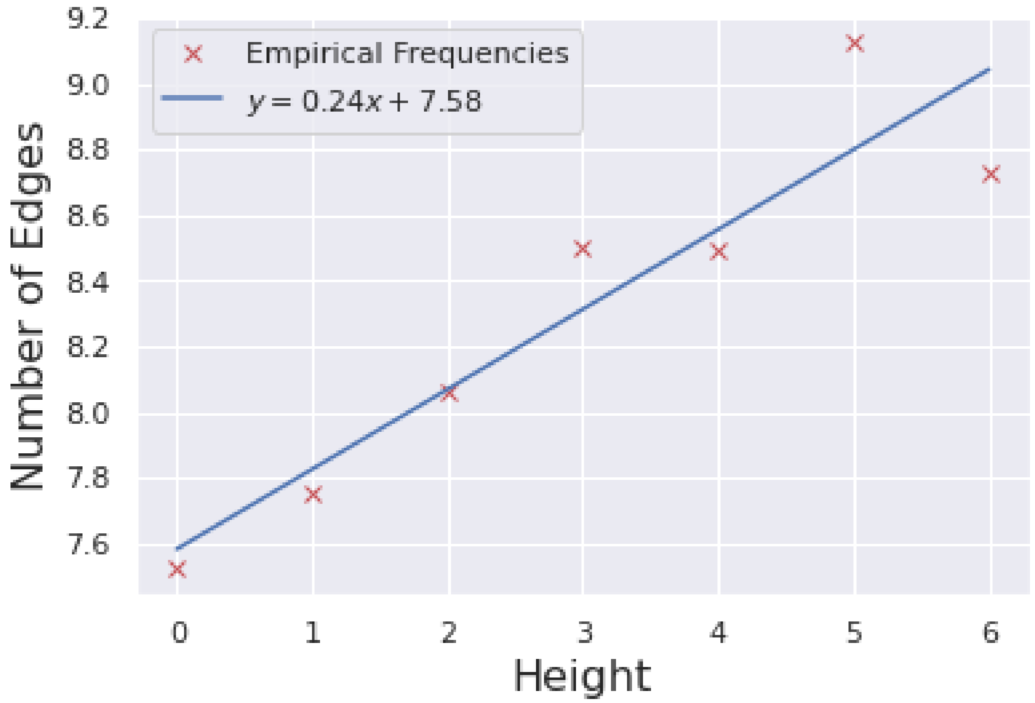

Supplementary Figure S10: Number of edges (log) with respect to height (linear) for the IGAM2 model with $H = 6,\ b = 3,\ c_1 = 1.5,\ c_2 = 2.5,\ H_0 = 2$. Empirical frequencies and linear fit are provided.

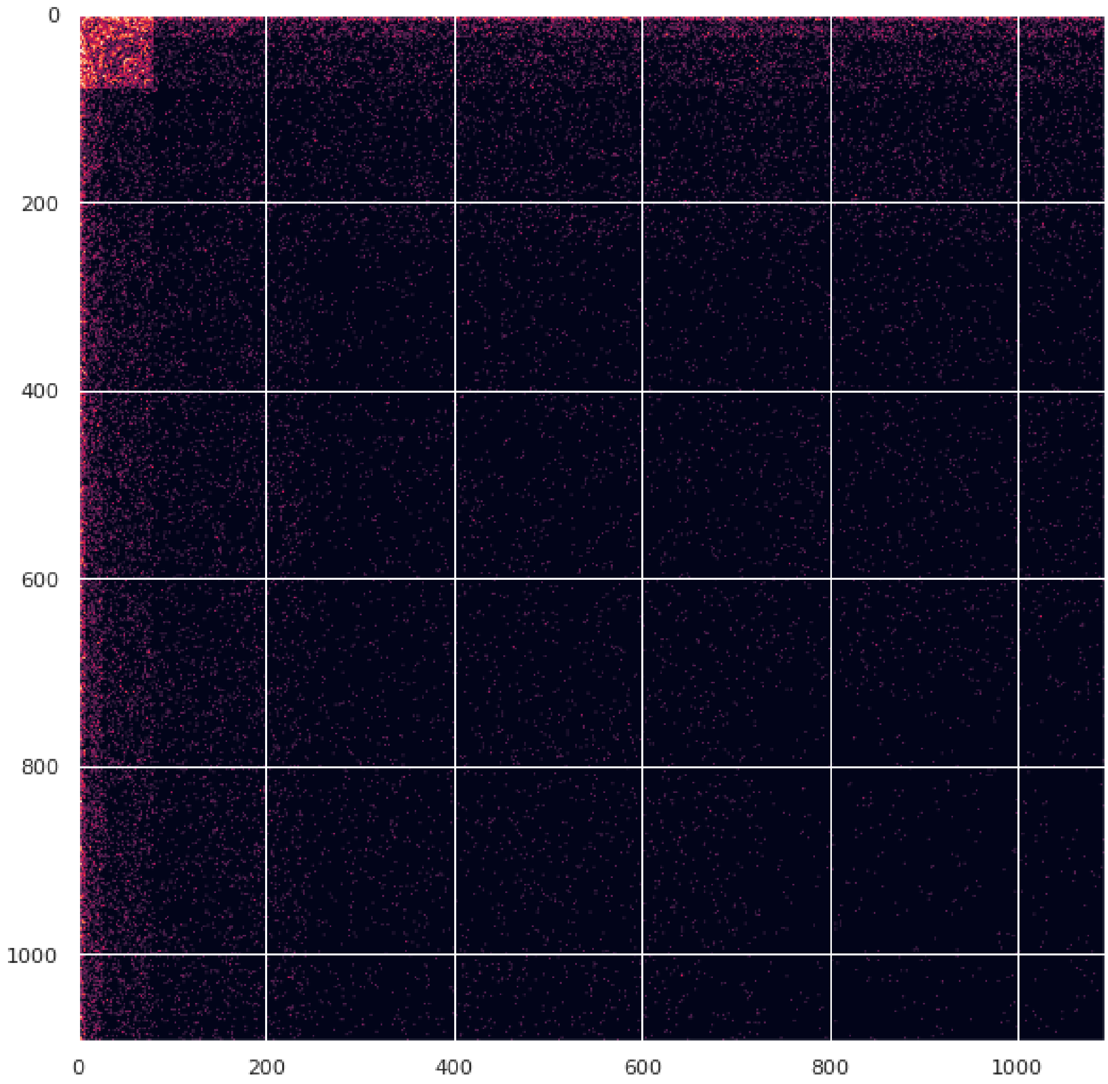

Supplementary Figure S11: Adjacency matrix for the IGAM2 model with $H = 6,\ b = 3,\ c_1 = 1.5,\ c_2 = 2.5,\ H_0 = 2$.

1.2 Average degree of IGAM

We provide plots of the average degree distribution of the IGAM model. We remind that the average degree at height h is given as

$$\begin{aligned}\bar{d}_h &\approx \sum_{r=0}^{H} b^r c^{-\min\{h,r\}-1} \\ &= \frac{1}{c}\left[\left(\frac{b}{c}\right)^{h+1} - 1 + \frac{b^{H+1} - b^{h+1}}{c^h}\right].\end{aligned}$$

We give a plot of $\log \bar{d}_h$ and observe that it is an almost linear function.

[ ]:
```
def plot_average_degree(H, b, c, new_figure=False, total_edges=False):

  h = np.arange(H + 1)

  d_h = 1 / c * ((b / c)**(h + 1) - 1 + (b**(H + 1) - b**(h + 1)) / c**h )
  if total_edges:
    d_h = d_h * b**h

  log_d_h = np.log(d_h)
  r2 = np.corrcoef(h, log_d_h)[0, 1]

  p = np.polyfit(h, log_d_h, deg=1)

  y = p[0] * h + p[1]
  if total_edges:
    hat_c = b * np.exp(-p[0])
  else:
    hat_c = np.exp(-p[0])

  if new_figure:
    plt.figure()
  plt.plot(h, log_d_h, linewidth=0, marker='x', label='Log average {} $b = {}, c␣
↪= {}$'.format('degree' if not total_edges else 'total edges', b, c))
  plt.plot(h, y, label='Linear approximation $y = {} h + {}, R^2 = {}, \hat c =␣
↪{}$'.format(round(p[0], 2), round(p[1], 2), round(r2, 3), round(hat_c, 2)))

  plt.xlabel('Height')
  plt.ylabel('Log average {}'.format('degree' if not total_edges else 'total␣
↪edges'))

  plt.legend(bbox_to_anchor=(1.04,1), loc="upper left")

plt.figure(figsize=(10, 10))
plot_average_degree(H=10, b=2, c=1.5)
plot_average_degree(H=10, b=3, c=2.5)
plot_average_degree(H=10, b=4, c=3.5)
plot_average_degree(H=10, b=20, c=3)

plt.figure(figsize=(10, 10))
plot_average_degree(H=10, b=2, c=1.5, total_edges=True)
plot_average_degree(H=10, b=3, c=2.5, total_edges=True)
plot_average_degree(H=10, b=4, c=3.5, total_edges=True)
plot_average_degree(H=10, b=20, c=3, total_edges=True)
```

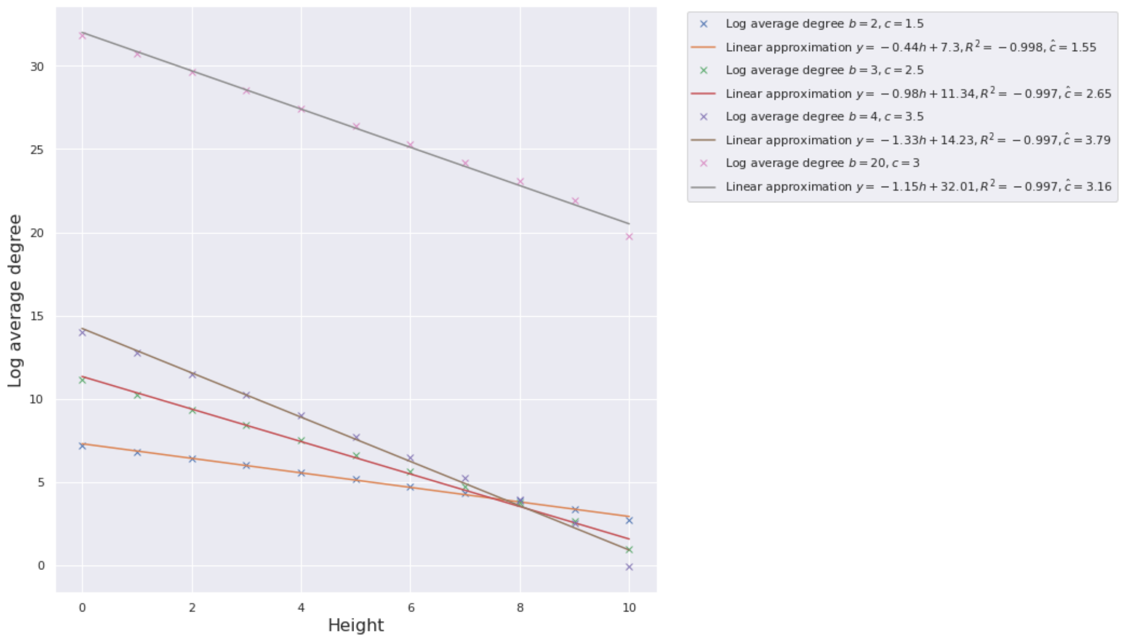

Supplementary Figure S12: Average degree (theoretical, log) with respect to height (linear) and linear fit approximation.

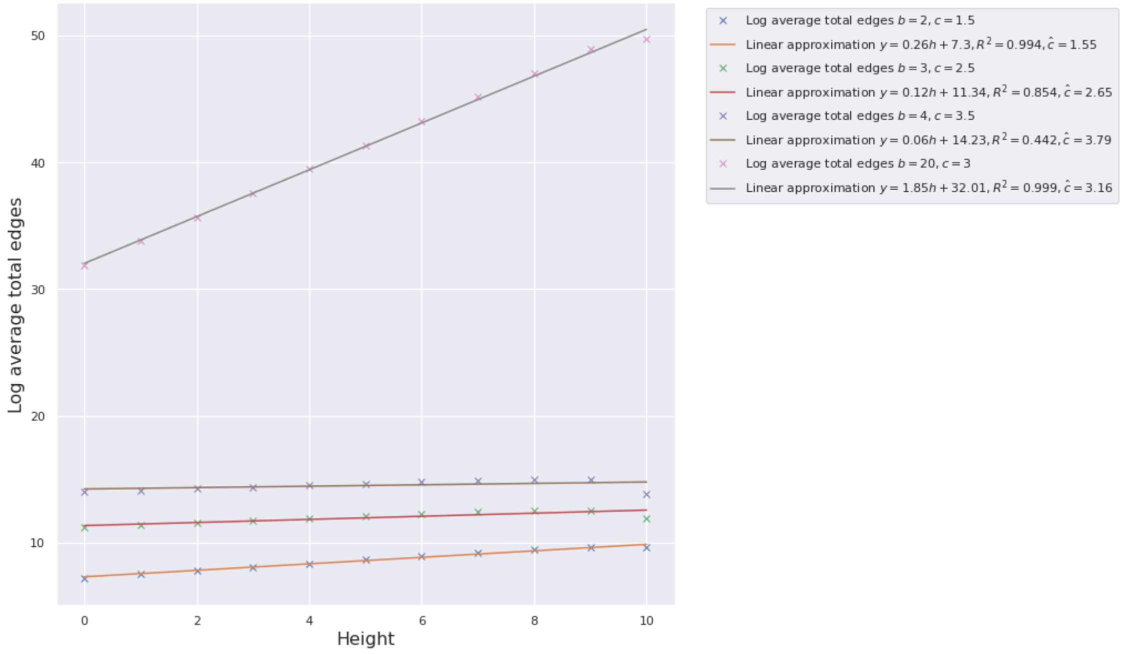

Supplementary Figure S13: Average number of edges (theoretical, log) with respect to height (linear) and linear fit approximation.

1.3 Fitting

We present the fitting algorithm for IGAM. The algorithm has $O(n^3)$ complexity. The algorithm's steps are as follows

1. The input is provided as a dataset of m edges $\mathcal{D} = \{e_1, \dots, e_m\}$.
2. Calculate the degree $\bar{y}_u$ of every node u in the sample.
3. We sort the degrees in descending order.
4. For all fanouts $b \in \{2, \dots, n-1\}$
5. We build a tree by attributing heights to the nodes in descending order of their degree
6. We calculate $\bar{z}_h = \log\left(\sum_{u:h(u)=h} \bar{y}_u\right)$, that is the log-total number of edges on level h as indicated by the samples.
7. We fit a linear least squares relation between h and $\bar{z}_h$ that has the form $\hat{z}_h = ah + b$
8. We calculate $c = b \cdot e^{-a}$, since the slope a is roughly $\log(b/c)$.

9. We calculate the likelihood of the parametrization which equals

$$\frac{1}{2}\sum_{u,v}\left(\mathbf{1}\{(u,v) \in \mathcal{D}\}\log(c^{-1-\min\{h(u),h(v)\}}) + (1 - \mathbf{1}\{(u,v) \notin \mathcal{D}\})\log(1 - c^{-1-\min\{h(u),h(v)\}})\right)$$

5. We return the set of parameters that maximize the computed likelihood.

[ ]:

```
# Fitting
def igam_fit(G, max_iters=-1):

  # Sample degree calculation
  degrees = list(sorted([(u, G.degree(u)) for u in G], key=lambda x: -x[1]))

  max_log_likelihood = - np.inf
  argmax_log_likelihood = (None, None, None)

  if max_iters <= 0:
    max_iters = len(G)

  for b in range(2, max_iters + 1):
    heights = {}
    h = 0
    i = 0
    # Give heights to everyone
    while i < len(G):
      for j in range(b**h):
        heights[degrees[i][0]] = h
        i += 1
        if i >= len(G):
           break
      h += 1
```

```
    # Calculate least squares fit for c
    frequencies = np.zeros(h)

    for u in G:
      frequencies[heights[u]] += G.degree(u)

    frequencies = frequencies / frequencies.sum()
    height_range = np.arange(len(frequencies))

    p = np.polyfit(height_range, np.log(frequencies), deg=1)

    a = p[0]

    c = b * np.exp(-a)

    if 1 < c < b:
      log_likelihood = 0

      for u in G:
        for v in G:
          if u != v:
            if G.has_edge(u, v):
              log_likelihood += (-1 - min(heights[u], heights[v])) * np.log(c)
            else:
              log_likelihood += np.log(1 - c**(-1 - min(heights[u], heights[v])))

      log_likelihood /= 2

      if log_likelihood >= max_log_likelihood:
        max_log_likelihood = log_likelihood
        argmax_log_likelihood = (b, c, h)

  return argmax_log_likelihood, max_log_likelihood
```

1.4 Fitting real-world data

We fit real world data. To be able to see the fits please change the path that the data are located. If the data is loaded successfully, you should be able to reproduce the plots of the paper.

[ ]:
```
def load_world_trade(location='datasets/world-trade/world-trade.csv'):
  df = pd.read_csv(location)
  G = nx.convert_matrix.from_pandas_edgelist(df, source='from', target='to')
  return G

def load_faculty(location='datasets/faculty/ComputerScience_edgelist.txt'):
  df = pd.read_csv(location, sep='\t')
```

```
  G = nx.convert_matrix.from_pandas_edgelist(df, source='# u', target='v')
  return G

def load_polblogs(location='datasets/polblogs/polblogs.mtx'):
  df = pd.read_csv(location, sep=' ', comment='%', header=None)
  G = nx.convert_matrix.from_pandas_edgelist(df, source=0, target=1)
  return G

def load_airports(location='data/airports/USairport500.txt'):
  df = pd.read_csv(location, sep=' ', header=None)
  G = nx.convert_matrix.from_pandas_edgelist(df, source=0, target=1)
  return G

def filter_nodes(G, threshold=4):
  filtered_nodes = []
  for v in G:
    if G.degree(v) <= threshold:
      filtered_nodes.append(v)
  G.remove_nodes_from(filtered_nodes)
  return G

def dominating_set(G, method='greedy'):
    x_axis = 1 + np.arange(len(G))
    y_axis = np.zeros_like(x_axis)
    n = len(G)

    if method == 'topk':
      degrees = list(sorted([(u, G.degree(u)) for u in G], key=lambda x: -x[1]))

    i = 0
    j = 0
    while len(G) > 0:
      if method == 'greedy':
        max_degree = 0
        current = None
        for u in G:
          if G.degree(u) >= max_degree:
            max_degree = G.degree(u)
            current = u
      elif method == 'topk':
        while not G.has_node(degrees[j][0]):
          j += 1
        current, _ = degrees[j]

      closed_neighborhood = [current] + [neigh for neigh in G[current]]
      y_axis[i] = n - len(G)
      G.remove_nodes_from(closed_neighborhood)
```

```
      i += 1

    y_axis[i:] = n
    y_axis = y_axis / n * 100
    x_axis = x_axis / n * 100

    return dominating_set, x_axis, y_axis

def fit_datasets(datasets):
  plt.figure(figsize=(10, 10))
  colors = iter(cm.rainbow(np.linspace(0, 1, len(datasets))))

  for G, name, max_iters in datasets:
    print('Name: {}, n = {}, m = {}'.format(name, len(G), len(G.edges())))
    argmax_log_likelihood, max_log_likelihood = igam_fit(G, max_iters=max_iters)
    degrees = list(sorted([(u, G.degree(u)) for u in G], key=lambda x: -x[1]))

    b, c, _ = argmax_log_likelihood
    color = next(colors)
    heights = {}
    h = 0
    i = 0
    # Give heights to everyone
    while i < len(G):
      for j in range(b**h):
        heights[degrees[i][0]] = h
        i += 1
        if i >= len(G):
          break
      h += 1

    # Calculate least squares fit for c
    frequencies = np.zeros(h)

    for u in G:
      frequencies[heights[u]] += G.degree(u)

    height_range = np.arange(len(frequencies))

    r2 = np.corrcoef(height_range, np.log(frequencies))[0, 1]

    p = np.polyfit(height_range, np.log(frequencies), deg=1)
    y = p[0] * height_range + p[1]

    plt.plot(height_range, np.log(frequencies), linewidth=0, marker='x', alpha=0.
↪7, c=color, label='{} ($b = {}, c = {}, LL = {}$)'.format(name, b, round(c,␣
↪2), round(max_log_likelihood, 2)))
```

```
    plt.plot(height_range, y, c=color, label='{} ($y = {} h + {}, R^2 = {}$)'.
 ↪format(name, round(p[0], 2), round(p[1], 2), round(r2, 2)))

  plt.legend(bbox_to_anchor=(1.04,1), loc="upper left")

  plt.ylabel('Degree Frequencies (log)')
  plt.xlabel('Height (linear)')

  plt.savefig('igam_fit.png')

  plt.figure(figsize=(10, 10))


  colors = iter(cm.rainbow(np.linspace(0, 1, len(datasets))))

  for G, name, _ in datasets:
    color = next(colors)
    _, x_axis_greedy, y_axis_greedy = dominating_set(G.copy(), method='greedy')
    _, x_axis_top_k, y_axis_top_k = dominating_set(G.copy(), method='topk')
    log_greedy = np.log10(y_axis_greedy)
    log_top_k = np.log10(y_axis_top_k)

    log_greedy = log_greedy[np.isfinite(log_greedy)]
    log_top_k = log_top_k[np.isfinite(log_top_k)]
    r2 = np.corrcoef(log_greedy, log_top_k)[0, 1]
    p = np.polyfit(log_greedy, log_top_k, deg=1)
    plt.plot(log_greedy, log_top_k, linewidth=0, c=color, alpha=0.7, marker='x',␣
 ↪label='{} ($R^2 = {}, \gamma = {}$)'.format(name, round(r2, 2), round(p[0],␣
 ↪2)))


  plt.legend()
  plt.xlabel('Percentage of dominated nodes using the greedy algorithm (log10)')
  plt.ylabel('Percentage of dominated nodes using the top-k nodes (log10)')
  plt.xlim(1.5, 2)
  plt.ylim(1.5, 2)

  plt.savefig('dominating_set.png')

datasets = [
  (filter_nodes(load_world_trade()), 'world-trade', -1),
  (filter_nodes(load_airports()), 'airports', -1),
  (filter_nodes(load_faculty()), 'cs-faculty', -1),
  (filter_nodes(load_faculty(location='data/faculty/History_edgelist.txt')),␣
 ↪'history-faculty', -1),
  (filter_nodes(load_faculty(location='data/faculty/Business_edgelist.txt')),␣
 ↪'business-faculty', -1),
```

```
  (filter_nodes(load_polblogs()), 'polblogs', -1)
]

fit_datasets(datasets)
```

```
Name: world-trade, n = 76, m = 845
Name: airports, n = 210, m = 2429
Name: cs-faculty, n = 205, m = 2861
Name: history-faculty, n = 145, m = 2334
Name: business-faculty, n = 113, m = 3027
Name: polblogs, n = 852, m = 15956
```

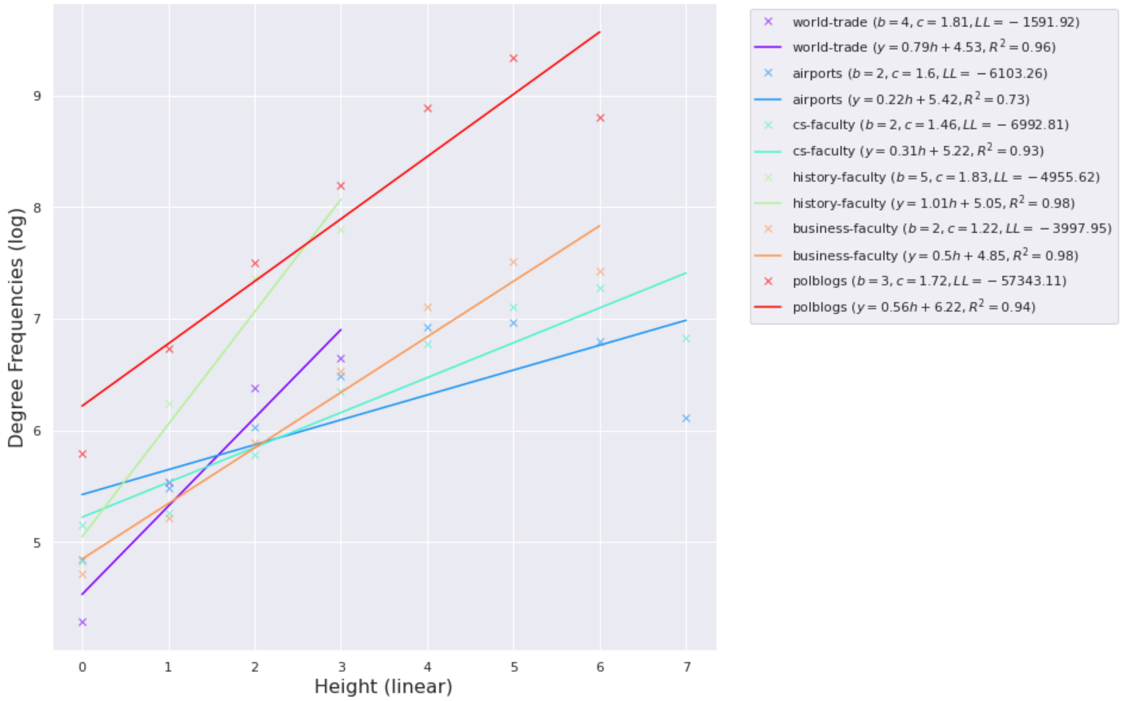

Supplementary Figure S14: Results of fitting an IGAM model to the world–trade, cs–faculty, history–faculty, business–faculty, and airports datasets examined in [5, 4, 2, 3, 1]. The Figure displays the predicted values of b and c for the IGAM model, and the total degree at each level h of the skeleton tree of fanout b. A linear fit is presented for each dataset to showcase the power law behaviour. Moreover values of the log–likelihood (LL), and Pearson's Correlation Coefficient R^2 are reported. Nodes with degree ≤ 4 have been filtered out as outliers.

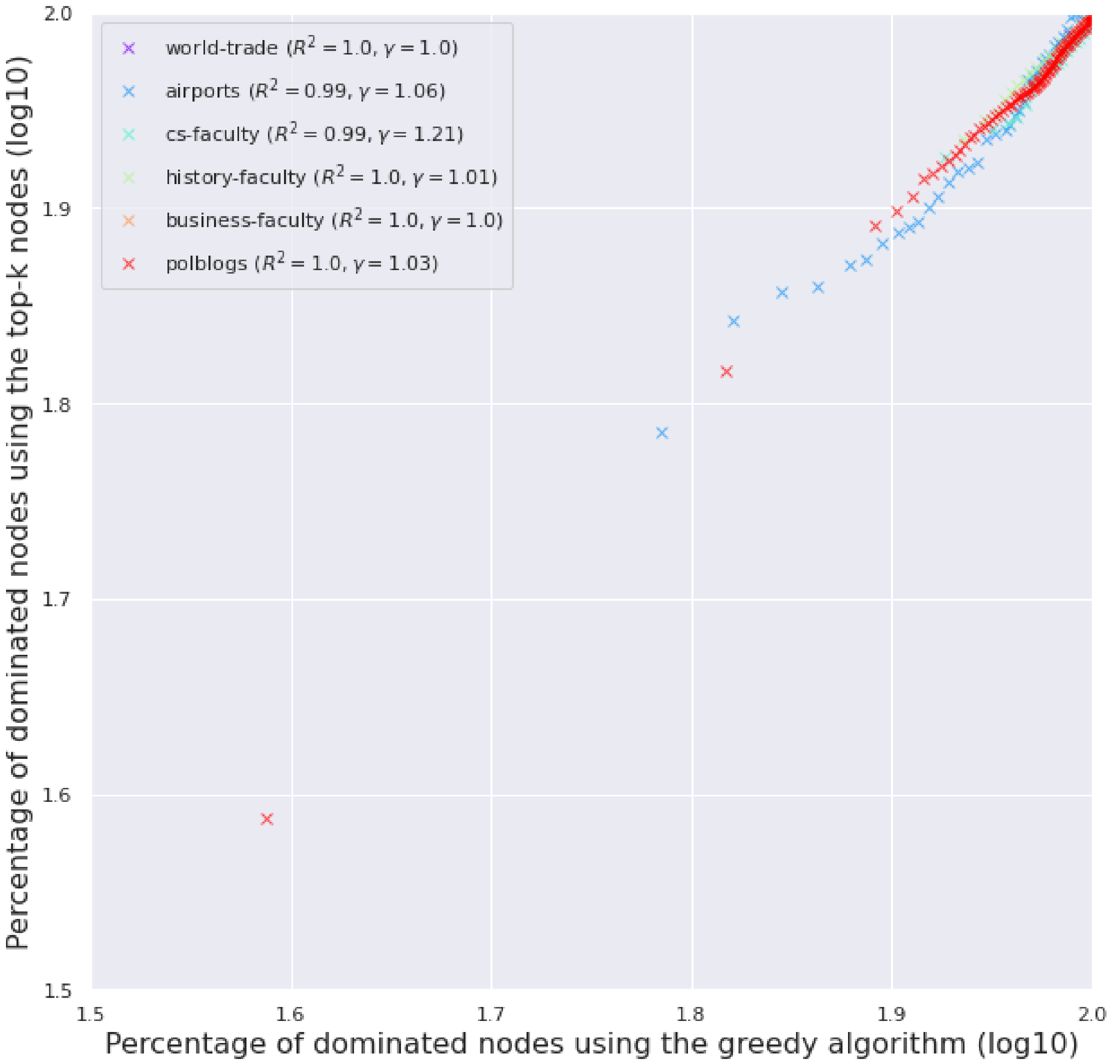

Supplementary Figure S15: Log–log plot between the percentages of dominated nodes when running the greedy $(1 - 1/e)$–maximum coverage algorithm of [6] (x–axis) and selecting nodes according to their hierarchy, i.e. in order of descending initial degree (y–axis). The slope γ and R^2 of linear fits are reported.

References

[1] Lada A Adamic and Natalie Glance. The political blogosphere and the 2004 us election: divided they blog. In *Proceedings of the 3rd international workshop on Link discovery*, pages 36–43, 2005.

[2] Aaron Clauset, Samuel Arbesman, and Daniel B Larremore. Systematic inequality and hierarchy in faculty hiring networks. *Science advances*, 1(1):e1400005, 2015.

[3] Vittoria Colizza, Romualdo Pastor-Satorras, and Alessandro Vespignani. Reaction–diffusion processes and metapopulation models in heterogeneous networks. *Nature Physics*, 3(4):276–282, 2007.

[4] Wouter De Nooy, Andrej Mrvar, and Vladimir Batagelj. *Exploratory social network analysis with Pajek: Revised and expanded edition for updated software*, volume 46. Cambridge University Press, 2018.

[5] Andrew Elliott, Angus Chiu, Marya Bazzi, Gesine Reinert, and Mihai Cucuringu. Core–periphery structure in directed networks. *Proceedings of the Royal Society A*, 476(2241):20190783, 2020.

[6] George L Nemhauser, Laurence A Wolsey, and Marshall L Fisher. An analysis of approximations for maximizing submodular set functions—i. *Mathematical programming*, 14(1):265–294, 1978.
